# A comparison of the efficacy of trastuzumab deruxtecan in advanced HER2-positive breast cancer: active brain metastasis versus progressive extracranial disease alone

**DOI:** 10.1016/j.esmoop.2023.102033

**Published:** 2023-10-20

**Authors:** J. Pearson, A. Khan, T. Bhogal, H. Wong, A. Law, S. Mills, N. Santamaria, J. Bishop, J. Cliff, D. Errington, A. Hall, C. Hart, Z. Malik, R. Sripadam, H. Innes, H. Flint, G. Langton, E. Ahmed, R. Jackson, C. Palmieri

**Affiliations:** 1 The Clatterbridge Cancer Centre NHS Foundation Trust, Liverpool; 2 Department of Molecular and Clinical Cancer Medicine, Institute of Systems, Molecular and Integrative Biology, University of Liverpool, Liverpool; 3 The Walton NHS Foundation Trust, Liverpool, UK

**Keywords:** breast cancer, HER2, trastuzumab deruxtecan, brain metastases

## Abstract

**Background:**

Trastuzumab deruxtecan (T-DXd) has demonstrated efficacy in patients with brain metastasis (BM), a group historically with poor outcomes. The prevalence of BMs in patients commencing T-DXd is currently unknown. No direct comparisons have been made of the activity of T-DXd in patients with active BM versus those with extracranial progression alone. This real-world study explored the prevalence of BMs in patients commencing T-DXd, the efficacy of T-DXd in active BM versus extracranial progression alone and the safety of T-DXd.

**Patients and methods:**

Patients with human epidermal growth factor receptor 2-positive advanced breast cancer treated with T-DXd between June 2021 and February 2023 at our specialist cancer hospital were identified and notes reviewed. Clinicopathological information, prior treatment, the presence or absence of central nervous system (CNS) disease, outcomes and treatment-emergent adverse events (TEAEs) were recorded.

**Results:**

Twenty-nine female patients, with a median age of 52 years (interquartile range 44-62 years), were identified; the prevalence of BM was 41%. Median number of lines of prior therapy was 2 (range 2-6). At a median follow-up of 13.8 months, median progression-free survival (PFS) for the overall population was 13.9 months [95% confidence interval (CI) 12.4 months-not estimable (NE)], 16.1 months (95% CI 15.1 months-NE) for active BMs and 12.4 months (95% CI 8.3 months-NE) for progressive extracranial disease alone. The 12-month overall survival (OS) rate was 74% (95% CI 59% to 95%) in the overall population, and 83% (95% CI 58% to 100%) and 66% (95% CI 45% to 96%) for active BMs and extracranial disease only, respectively. Most common TEAEs were fatigue, alopecia, and constipation. In nine patients (31%, including two deaths), pneumonitis occurred.

**Conclusion:**

In this real-world population, we demonstrate T-DXd to be effective in patients with active BMs and those with progressive extracranial disease alone. PFS and OS were numerically longer in those with active BMs. These data demonstrate that patients with active BM treated with T-DXd have at least comparable outcomes to those with extracranial disease alone. The high rate of pneumonitis warrants further consideration.

## Highlights

• The prevalence of BM in a contemporaneous real-world population commencing T-DXd was found to be 41%.
• Median PFS: 16.1 months (95% CI 15.1-NE) active BM vs 12.4 (95% CI 8.3-NE) progressive extracranial disease.
• Median OS: 15.3 months (95% CI 15.1-NE) active BM vs 13.4 (95% CI 8.3-NE) for progressive extracranial disease.
• The rate of pneumonitis was 31%.

## Introduction

A clinical feature of metastatic human epidermal growth factor receptor 2-positive (HER2-positive) breast cancer (BC), irrespective of the estrogen receptor (ER) status, is a predilection for the central nervous system (CNS).^1^ Data from the era of adjuvant trastuzumab found that while the CNS is an uncommon site of initial relapse in HER2-positive BC, up to 47% have evidence of CNS disease at the time of death.^2^ More recent real-world data have found that at 24 months after diagnosis of metastatic disease, the cumulative incidence rate of CNS metastases was 29.2% [95% confidence interval (CI) 26.8% to 31.8%] and 49.0% (95% CI 45.7% to 52.5%) in patients with HER2-positive/hormone receptor-positive and HER2-positive/hormone receptor-negative BC, respectively.^3^ The development of brain metastases (BMs) has been associated with a worse overall survival (OS) and quality of life compared to those without BMs in patients with HER2-positive metastatic BC.^4^^,^^5^ The lack of effective systemic treatment options in patients with BM and HER2-positive disease after the failure of local treatments resulted in a number of studies with novel agents to address this unmet need.

HER2CLIMB, which compared the activity of tucatinib in combination with trastuzumab and capecitabine versus placebo in combination with trastuzumab and capecitabine, was the first phase III study to actively recruit patients with BM including those with active BM, and included protocol-defined intracranial endpoints.^6^^,^^7^ It demonstrated an improvement in intracranial progression-free survival (PFS) from 4.2 to 9.9 months and a 42% reduction of risk of death [hazard ratio (HR) 0.58, 95% CI 0.40-0.85] for tucatinib in patients with BM.^7^

The activity of the second-generation antibody drug conjugate (ADC) trastuzumab deruxtecan (T-DXd) with regard to BM has been assessed in two single-arm phase II studies, TUXEDO and DEBBRAH.^8^^,^^9^ In both studies the primary endpoint was intracranial objective response rates (ORRs), as defined by response assessment in neuro-oncology brain metastases (RANO-BM).^8^^,^^9^ TUXEDO and DEBBRAH recruited 15 and 9 patients, respectively; 9 patients in both studies had progressive intracranial disease despite previous local and systemic therapy, with the remaining 6 in TUXEDO having newly diagnosed and untreated disease.^8^^,^^9^ Intracranial ORRs of 44% and 73% were reported in the intention-to-treat population for DEBBRAH and TUXEDO, respectively.^8^^,^^9^ DESTINY-Breast02 and 03 have, respectively, demonstrated the superiority of T-DXd to physician’s choice and trastuzumab emtansine (T-DM1).^10^^,^^11^ Both these studies only allowed patients with stable BM and demonstrated meaningful improvements in outcomes with T-DXd in this group although neither study had formal intracranial endpoints.

A number of important questions remain unanswered in regard to T-DXd. These include the prevalence of BM in a contemporaneous population commencing T-DXd and the outcomes of patients with active BM treated with T-DXd compared to those with active/progressive extracranial disease alone.

Given this and the historical data regarding the poorer outcomes of patients with HER2-positive BM disease, we sought to explore the real-world clinical outcomes of patients with and without active BM treated with T-DXd at a tertiary referral cancer hospital in the UK.

## Patients and methods

### Patient population

Patients with HER2-positive locally advanced or metastatic BC treated with T-DXd as part of their routine care from 1 April 2021 until 28 February 2023 at The Clatterbridge Cancer Centre NHS Foundation Trust were identified via the central pharmacy records. Patients were grouped by the presence or absence of CNS metastases and the nature of that CNS disease, BM or leptomeningeal disease (LMD). Those receiving at least one cycle of T-DXd were assessed.

The population for purposes of analysis was defined as follows: (i) the overall cohort; (ii) those with known extracranial disease alone; (iii) the BM group, which was divided into those with stable disease versus active BM defined as radiological progression at commencement of T-DXd following local therapy or newly diagnosed untreated BM; and (iv) LMD.

The project was approved as an audit by the local clinical audit subcommittee (ref number 2122-45); therefore, informed consent was not required.

### Data collection and analysis

Clinicopathological data collected included: age, sex, performance status, tumour information including the ER status and sites of metastasis. Prior number of lines of HER2 therapy and nature of these in the metastatic setting as well as any prior local CNS treatment were also collected.

### Clinical data collected and definitions

Objective response was defined as a confirmed complete response or partial response according to the RECIST 1.1 criteria. Clinical benefit was defined as a complete response, a partial response or stable disease for ≥24 weeks. PFS was defined as the time from first infusion of T-DXd until the date of radiologically documented progression or death. OS was measured from the date of first dose of T-DXd to death from any cause. Only patients with baseline radiology and subsequent post-treatment scans were used in ORR; however, those without were used in PFS and OS. Those who did not experience an event were censored at the date of last being seen alive. Data cut-off was 28 February 2023.

### Treatment-emergent adverse events

Treatment-emergent adverse events (TEAEs) were measured by type, incidence and severity using the Common Terminology Criteria for Adverse Events (CTCAE) V5.0. These were recorded by nursing staff, before each cycle of treatment, and were abstracted from electronic medical records. Adverse events of special interest were defined as pneumonitis and radionecrosis.

### Statistical analysis

Continuous variables are presented with their median and interquartile range (IQR), while categorical variables are described as frequency counts and proportion percentages. Statistical analysis was carried out with R version 4 (Bell Laboratories, Murray Hill, NJ).

## Results

### Baseline characteristics

Thirty female patients were commenced on T-DXd between 1 April 2021 and 28 February 2023. One patient was commenced on T-DXd for radiological progression in the chest; a subsequent biopsy of the lesion demonstrated non-small-cell lung cancer and the patient was excluded from the analysis. Of the remaining 29 patients, 41% (12 of 29) had BMs of which 83% (10 of 12) were active, 8% (1 of 12) stable and 8% (1 of 12) did not have a baseline scan to compare to and therefore status was unknown and hence not grouped. There was one (3%) case of LMD; of note, the patient had previously demonstrated metastatic disease that was both HER2 negative and HER2 positive. Therefore, 45% of patients (13 of 29) had CNS disease at commencement of T-DXd. Fifty-five percent (16 of 29) had extracranial disease alone, which was progressive in all cases. In those with BM, extracranial disease was progressive in 75% (9 of 12) and BM was the sole site of progression in 25% (3 of 12).

Baseline clinicopathological information is displayed in Table 1. Median age was 52 years (IQR 44-62 years) for the overall population, 50 years (IQR 43-53 years) for the BM group and 54 years (IQR 50-67.5 years) for the extracranial group. Thirty-eight percent (11 of 29) of all patients had presented with *de novo* disease. The proportion with *de novo* disease was higher for those with BM, 50% (6 of 12) as compared to 31% (5 of 16) for those with extracranial disease alone. Seventy-six percent (22 of 29) of patients had ER-positive disease. Of note, 17% (5 of 29) were defined as HER2 positive following a biopsy of metastatic disease, having previously had documented HER2-negative-based primary BC.

**Table 1:** Clinicopathological characteristics

Patient characteristics	All (*n* = 29)	Extracranial cohort (*n* = 16)	CNS (*n* = 13)	Total BM cohort (*n* = 12)	LMD (*n* = 1)
Sex, *n* (%)					
Female	29 (100)	16 (100)	13 (100)	12 (100)	1 (100)
Median age, years (IQR)					
Age at baseline	52 (44-62)	54 (50-67.5)	50 (42.5-53)	50 (43-53)	42 (42)
*De novo* presentation, *n* (%)					
Yes	11 (38)	5 (31)	6 (46)	6 (50)	0 (0)
No	18 (62)	11 (69)	7 (54)	6 (50)	1 (100)
Primary disease HER2 negative, *n* (%)^a^	5 (17)	4 (25)	1 (8)	1 (8)	—
Estrogen receptor status, *n* (%)					
Positive	22 (76)	13 (81)	9 (69)	8 (67)	1 (100)
Negative	7 (24)	3 (19)	4 (31)	4 (33)	0 (0)
ECOG performance status at baseline, *n* (%)					
0	8 (28)	5 (31)	3 (23)	3 (25)	0 (0)
1	21 (72)	11 (69)	10 (77)	9 (75)	1 (100)
Pregnant at diagnosis of MBC, *n* (%)	2 (7)	1 (6)	1 (8)	—	1 (100)
Median time since diagnosis of MBC to commencement of T-DXd, months (IQR)	39.0 (16.8-65.9)	30.5 (16.8-54.4)	52.6 (19.2-67.1)	57.8 (19.2-67.1)	39
Number of prior lines of treatment for MBC, median (range)					
HER2 directed and chemotherapy	2 (2-6)	2 (2-4)	2 (2-6)	2 (2-6)	2 (2)
Prior HER2 directed and chemotherapy, *n* (%)					
Trastuzumab, taxane and pertuzumab	25 (86)	14 (88)	11 (85)	11 (92)	—
Trastuzumab emtansine	29 (100)	16 (100)	13 (100)	12 (100)	1 (100)
Capecitabine	4 (14)	2 (13)	2 (15)	2 (17)	—
Eribulin	4 (14)	2(13)	2 (15)	2 (17)	—
Capecitabine and neratinib	3 (10)	2 (13)	1 (8)	1 (8)	—
Paclitaxel	2 (7)	1 (6)	1 (8)	1 (8)	—
Epirubicin and cyclophosphamide	1 (3)	1 (6)	—	—	—
Fluorouracil, epirubicin and cyclophosphamide	1 (3)	1 (6)	—	—	—
Paclitaxel plus gemcitabine	1 (3)	—	1 (8)	1 (8)	—
Trastuzumab duocarmazine	1 (3)		1 (8)	1 (8)	—
Trastuzumab, pertuzumab, letrozole and palbociclib^b^	1 (3)	—	1 (8)	—	1 (100)
Site of progressive disease, *n* (%)					
Extracranial and cranial	8 (28)	—	8 (62)	7 (58)	1 (100)
Extracranial only	18 (62)	16 (100)	2 (15)	2 (17)	—
Cranial only	3 (10)	—	3 (23)	3 (25)	—
No. of prior local therapies for BMs, *n* (%)					
None	1 (3)	—	1 (8)	1 (8)	—
1	9 (31)	—	9 (69)	9 (75)	—
2	2 (7)	—	2 (15)	2 (17)	—
Type of prior therapy for BMs (if given), *n* (%)					
Resection	1 (3)	—	1 (8)	1 (8)	
Stereotactic radiosurgery	4 (14)	—	4 (31)	4 (33)	
Whole-brain radiotherapy	8 (28)	—	8 (62)	8 (67)	

BM, brain metastasis; CNS, central nervous system; ECOG, Eastern Cooperative Oncology Group; HER2, human epidermal growth factor receptor 2; IHC, immunohistochemistry; IQR, interquartile range; LMD, leptomeningeal disease; MBC, metastatic breast cancer; T-DXd, trastuzumab deruxtecan.

a Biopsy of metastatic disease demonstrated HER2 positivity.

b Primary breast cancer was HER2 positive, subsequently two separate assessments of metastatic sites: (1) ovaries: HER2 positive (IHC 2+, FISH+) and (2) vertebrae FISH: HER2 negative. Single-agent paclitaxel during pregnancy, with trastuzumab, letrozole and palbociclib commenced postpartum.

Both the BM and extracranial alone groups had received a median of two lines of prior HER2 therapy and chemotherapy (Table 1). Eighty-six percent (25 of 29) had received trastuzumab plus pertuzumab and 100% (29 of 29) T-DM1. One patient had received prior treatment with T-DM1 and trastuzumab duocarmazine. Prior endocrine therapy for metastatic disease in ER-positive patients is summarised in Supplementary Table S1, available at https://doi.org/10.1016/j.esmoop.2023.102033.

With regard to local CNS therapy, 8% (1 of 12) had undergone surgical resection, 33% (4 of 12) stereotactic radiosurgery (SRS), 67% (8 of 12) whole-brain radiotherapy (WBRT) and 8% (1 of 12) SRS with subsequent WBRT. Only 8% (1 of 12) of patients had received no local CNS treatment for their BM. Therefore, 92% (11 of 12) of BM patients had received at least one prior local therapy.

The median time from diagnosis of metastatic disease to treatment with T-DXd for the overall population was 39.0 months (IQR 16.8-65.9 months). For the BM and extracranial alone groups, this was 57.8 months (IQR 19.2-67.1 months) and 30.5 months (IQR 16.8-54.4 months), respectively. The time from diagnosis of BM to T-DXd treatment was 14.4 months (IQR 2.8-31.5 months).

### Efficacy data

At a median follow-up time of 13.8 months (95% CI 11.1-17.0 months), the median PFS for the overall population was 13.9 months [95% CI 12.4 months-not estimable (NE)] (Table 2). The median PFS for the BM group was 17.0 months (95% CI 15.2 months-NE) and for those with active BM 16.1 months (95% CI 15.1 months-NE). While the median PFS in the extracranial disease alone group was 12.4 months (95% CI 8.3 months-NE) (*P* = 0.106 against active disease), PFS was 0.3 months for the sole case of LMD. The 12-month PFS for the overall population was 69% (95% CI 53% to 92%), while in the total BM group, the 12-month PFS was at 88% (95% CI 67% to 100%), and 83% (95% CI 58% to 100%) in those with active BMs. For those with extracranial disease alone, the 12-month PFS was 56% (95% CI 35% to 91%) (Table 2).

**Table 2:** Efficacy data: progression-free survival, overall survival and objective response data to trastuzumab deruxtecan

Outcome	Overall (*n* = 29)	Extracranial cohort (*n* = 16)	CNS (*n* = 13)	Total BM (*n* = 12)	Active BM (*n* = 10)	LMD (*n* = 1)
No. of progressions, *n* (%)^a^	15 (52)	9 (56)	6 (46)	5 (42)	5 (50)	1 (100)
Median PFS, months (95% CI)	13.9 (12.4-unobtainable)	12.4 (8.3-unobtainable)	15.2 (15.1-unobtainable)	17.0 (15.2-unobtainable)	16.1 (15.1-unobtainable)	0.30
12-month PFS, % (95% CI)	69 (53-92)	56 (35-91)	81 (60-100)	88 (67-100)	83 (58-100)	—
Deaths, *n* (%)	11 (38)	7 (44)	4 (31)	3 (25)	3 (30)	1 (100)
Median OS, months, % (95% CI)	15.3 (12.6-NE)	13.4 (8.3-NE)	15.3 (15.1-NE)	NR (15.3-NE)	15.3 (15.1-NE)	0.3
12-month OS, % (95% CI)	74 (59-95)	66 (45-96)	81 (60-100)	88 (67-100)	83 (58-100)	—
Confirmed objective response rate, % (95% CI)^a^	69 (52-86)	69 (46-91)	54 (27-81)	58 (30-86)	60 (30-90)	0 (0)
Brain metastases						
Partial response, *n* (%)	7 (54)	—	7 (54)	7 (58)	6 (60)	—
Stable disease, *n* (%)	3 (23)	—	3 (23)	3 (25)	3 (30)	—
Progressive disease, *n* (%)	—	—	—	—	—	—
Not evaluable, *n* (%)^b^	3 (23)	—	3 (23)	2 (17)	1 (10)	1 (100)
Disease control rate, % (95% CI)	77 (54-100)	—	77 (54-100)	83 (62-100)	90 (71-100)	—
Extracranial metastases						
Partial response, *n* (%)	16 (55)	11 (69)	5 (38)	5 (42)	3 (30)	—
Stable disease, *n* (%)	8 (28)	2 (13)	6 (46)	6 (50)	6 (60)	—
Progressive disease, *n* (%)^c^	1 (3)	1 (6)	—	—	—	—
Not evaluable, *n* (%)^b^	4 (14)	2 (13)	2 (15)	1 (8)	1 (10)	1 (100)
Disease control rate, % (95% CI)	83 (69-97)	81 (62-100)	85 (65-100)	92 (77-100)	90 (71-100)	—

BM, brain metastasis; CI, confidence interval; CNS, central nervous system; LMD, leptomeningeal disease; NE, not estimable; ORR, objective response rate; OS, overall survival; PFS, progression-free survival; T-DXd, trastuzumab deruxtecan.

a Confirmed ORR for the overall population was taken as the best response from extracranial and intracranial disease combined. For the extracranial cohort, the recorded ORR was best response from assessing solely extracranial disease, and for CNS disease it was from assessing solely intracranial disease.

b There were five patients who were not assessable radiologically (two extracranial, two BM and one LMD) due to death (*n* = 3), toxicity (*n* = 1) before reimaging and lack of baseline comparator (*n* = 1). These patients were not assessable for ORR.

c Two patients in the BM group continued T-DXd following small-volume asymptomatic intracranial progression. In one case this occurred following 2 months of treatment interruption due to thrombocytopenia.

Radiological assessment of response was not available for five patients due to lack of baseline imaging (one case), repeat imaging not carried out due to death (three cases) or discontinuation of treatment for toxicity (one case). Combined intracranial and extracranial ORR in the overall cohort was 69% (20 of 29; 95% CI 52% to 86%) (Table 2). In the total BM group, the intracranial ORR was 58% (7 of 12; 95% CI 30% to 86%) and 60% (6 of 10; 95% CI 30% to 90%) for those with active BMs. ORR was 69% (11 of 16; 95% CI 46% to 91%) for those with extracranial disease alone. The clinical benefit rate was 83% (95% CI 62% to 100%) in the BM group and 90% (95% CI 71% to 100%) for those with active BM. A clinical benefit rate of 81% (95% CI 62% to 100%) was recorded for those with extracranial disease alone (Table 2). Progressive disease was the best response for the single case of LMD.

The median OS for the overall population was 15.3 months (95% CI 12.6 months-NE). With the median OS unobtainable for the total BM group (95% CI 15.3 months-NE), it was 15.3 months (95% CI 15.1 months-NE) for active BMs as compared to 13.4 months (95% CI 8.3 months-NE) for the extracranial disease alone group. Twelve-month OS estimates were 74% (95% CI 59% to 95%) for the overall population, 88% (95% CI 67% to 100%) for the total BM group and 83% (95% CI 58% to 100%) for active BMs. In patients with extracranial disease alone, it was 66% (95% CI 45% to 96%).

At data cut-off, 18 of 29 (62%) patients were alive. Forty-eight percent (14 of 29) remain on T-DXd: 58% (7 of 12) total BM, 50% (5 of 10) active BM and 44% (7 of 16) extracranial disease alone. Of the 15 (52%) who had discontinued treatment, 10 were due to progressive disease or deaths and 5 due to treatment-related toxicity (Supplementary Table S2, available at https://doi.org/10.1016/j.esmoop.2023.102033).

There were seven deaths in the extracranial group (reasons: four due to disease progression, two pneumonitis, one non-neutropenic sepsis) and four in the CNS group, three in the BM cohort and one LMD, all due to disease progression.

### Safety

All patients were assessed for drug-related TEAEs, and all developed at least one TEAE of any grade, with 31% (9 of 29) being grade 3 or higher. The three most common toxicities were fatigue (97%; 28 of 29), alopecia (76%; 22 of 29) and constipation (72%; 21 of 29) (Table 3). TEAEs resulted in ≥1 dose reductions in 41% (12 of 29). Dose interruptions, reductions and discontinuations and reasons are summarised in Supplementary Table S2, available at https://doi.org/10.1016/j.esmoop.2023.102033.

**Table 3:** Treatment-emergent adverse events of any grade which occurred in >25% of patients, all grade ≥3 and all those of special interest

Adverse event, *n* = 29 patients	Any grade, *n* (%)	Grade ≥3, *n* (%)
**Any adverse event**		
Fatigue	28 (97)	0
Alopecia	22 (76)	0
Constipation	21 (72)	0
Peripheral neuropathy	20 (69)	0
Anaemia^a^	19 (86)	0
Nausea	18 (62)	1 (3)
Leukopenia^a^	14 (64)	1 (5)
Diarrhoea	13 (45)	2 (7)
Neutropenia^a^	12 (55)	0
Aspartate aminotransferase increased^a^	12 (55)	2 (9)
Alanine aminotransferase increased^a^	10 (45)	0
γ-Glutamyltransferase^a^	10 (45)	0
Infection	9 (31)	0
Vomiting	7 (24)	0
Thrombocytopenia^a^	6 (27)	0
**Adverse events of special interest**		
Pneumonitis	9 (31)	3 (10)
Radionecrosis	1 (3)	—

a Blood results, including haematology and biochemistry, were only available from 22 patients and the percentage displayed has been changed accordingly.

With regard to events of special interest, 31% (9 of 29) developed pneumonitis and 3% (1 of 29) developed radionecrosis (Table 3). With regard to pneumonitis, 66.6% (6 of 9) were grade 1, 11.1% (1 of 9) grade 3 and 22.2% (2 of 9) grade 5 death events; the outcomes of the episodes of pneumonitis are summarised in Supplementary Table S3, available at https://doi.org/10.1016/j.esmoop.2023.102033. Radionecrosis was diagnosed pathologically and surgically debrided. Treatments given post-progression after T-DXd are summarised in Supplementary Table S4, available at https://doi.org/10.1016/j.esmoop.2023.102033.

## Discussion

In the current study, we present real-world data on a cohort of patients with advanced HER2-positive BC commencing on T-DXd at a specialist cancer centre. T-DXd was initially made available in England in April 2021 for patients with locally advanced or metastatic disease who had received two or more lines of HER2-directed therapy. We present data on the prevalence of BM in a contemporaneous population, the activity of T-DXd in patients with and without active BM as well as real-world safety data.

The prevalence of known CNS disease (BM/LMD) and BM in patients commencing T-DXd was 45% (13 of 29) and 41% (12 of 29), respectively. No contemporaneous data relating to the prevalence of BM in a population commencing T-DXd are currently available. DESTINY-Breast02 and 03 both mandated cross-sectional imaging of the brain before study entry, excluding patients with untreated or progressive BM (Supplementary Table S5, available at https://doi.org/10.1016/j.esmoop.2023.102033).^10^^,^^11^ However, the proportion failing screening based on protocol-defined BM criteria has not been reported. Data from registHER, which followed patients with newly diagnosed HER2-positive metastatic disease between 2003 and 2006, reported that 21% of patients had developed CNS disease by the third line of metastatic treatment.^4^ An indirect comparison with our data does suggest that CNS disease is more common in patients reaching third-line treatment in the current era of HER2 therapy. Given the current lack of routine screening of the CNS, the currently reported BM rate may actually be an underestimation.

The diagnosis of new BM in those with extracranial disease alone at the commencement of T-DXd was uncommon, occurring in 6% (1 of 16) at a median follow-up of 12.9 months (95% CI 10.6 months-NE). This represented 14% (1 of 7) of all progression events. DESTINY-Breast01 reported that 1.3% (2 of 160) of patients with no known CNS disease developed progression within the CNS on T-DXd.^12^ DESTINY-Breast02 and 03 did not report on the development of new BM in those with extracranial disease alone at study entry or the proportion of progression events that were CNS; therefore, a comparison to our data is not possible.^10^^,^^11^

In our overall population, the combined intracranial and extracranial ORR was 69% (95% CI 52% to 86%) with a median PFS of 15.3 months (95% CI 12.4 months-NE). These data confirm the activity of T-DXd and extend the data reported in previous studies to a more diverse population that is more reflective of the real world namely patients progressing extracranially and intracranially.^10^^,^^11^ Our reported median PFS in those with extracranial disease only, at 12.4 months (95% CI 8.3 months-NE), is less than that reported in the extracranial only group within DESTINY-Breast02 at 18.7 months (95% CI 15.1-24.8 months).^10^

Ten patients within our study had active BM making up 83.3% (10 of 12) of the total BM population, compared to 9-15 active BM cases that formed the entire population for TUXEDO and DEBBRAH^8^^,^^9^ (Supplementary Table S6, available at https://doi.org/10.1016/j.esmoop.2023.102033). A recent US real-world study reported on 10 patients with active BM, forming 56% of their reported population^13^ (Supplementary Table S6, available at https://doi.org/10.1016/j.esmoop.2023.102033). The median follow-up time for our BM population was 13.8 months (95% CI 9.9 months-NE) as compared to 7-12 months for the prior studies^8^^,^^9^^,^^13^ (Supplementary Table S6, available at https://doi.org/10.1016/j.esmoop.2023.102033), with all patients in the current report having received T-DM1, as compared to 60% for TUXEDO-1, 67% for DEBBRAH and 83% for the US real-world study.^8^^,^^9^^,^^13^ The median number of lines of prior treatment for metastatic disease in our population at 2 was comparable to TUXEDO but fewer than in both DEBBRAH and the US real-world study^8^^,^^9^^,^^13^ (Supplementary Table S6, available at https://doi.org/10.1016/j.esmoop.2023.102033).

For our active BM group, we report an ORR, as defined by RECIST, of 60% (95% CI 30% to 90%), with a PFS of 16.1 months (95% CI 15.1 months-NE) and 12-month PFS of 83% (95% CI 58% to 100%). While cross-study comparisons can be difficult, particularly when using different response criteria, the ORR for our active BM group compares favourably to ORR, defined by RANO-BM, within TUXEDO which had a similar number of prior lines of treatment.^8^ The greater ORR in comparison to DEBBRAH likely reflects the more heavily pre-treated population within that study,^9^ while the higher ORR within the US real-world study^13^ may be related to the lower proportion with active BMs and the higher proportion with untreated and stable BM compared to our population (Supplementary Table S6, available at https://doi.org/10.1016/j.esmoop.2023.102033).

The median PFS of 16.1 months (95% CI 15.1 months-NE) within our active BM group is similar to TUXEDO (14 months; 95% CI 11.0 months-NE), while PFS has yet to be reported by DEBBRAH^9^ and was not reached within the US real-world study.^13^ We report a median OS of 15.3 months (95% CI 15.1 months-NE) and a 12-month OS of 83% (95% CI 58% to 100%) for our active BM group. Median OS was not reached within TUXEDO, with three deaths at a median follow-up of 12 months.^8^ Therefore, at 12 months a similar proportion are alive with active BM in our cohort and TUXEDO. DEBBRAH and the real-world US study have yet to report OS data,^9^^,^^13^ while DESTINY-Breast02 has not reported OS in those with stable BM.^10^ The estimated 1-year OS within HER2CLIMB for the tucatinib combination group with active BM was 70.7% (95% CI 61.5% to 78.1%) as compared to 46.4% (95% CI 33.1% to 58.8%) for the placebo combination group.^14^ Overall, all these data demonstrate the clinical benefits of modern HER2 therapies in patients with active BM. Accepting the differences between the prior studies, our data in a population predominately dominated by active BM confirm that T-DXd has intracranial activity that compares favourably with the previous studies.^8^^,^^9^^,^^13^

There have been no reports on the efficacy and outcome of patients with active BM versus those with extracranial progression alone treated with T-DXd. We report a median PFS in active BM of 16.1 months (95% CI 15.1 months-NE), versus 12.4 months (95% CI 8.3 months-NE) for the extracranial disease alone, while the median OS was 15.3 months (95% CI 15.1 months-NE) versus 13.4 months (95% CI 8.3 months-NE) for those with active BM and extracranial disease alone, respectively. These data suggest that the outcomes of patients with active BM are not inferior to those of patients with extracranial progression, given that due to the nature of the study we lack power to detect a statistically significant difference. registHER, a registry study in the trastuzumab era, demonstrated that the development of BM was a detriment to survival, with a median OS of 26.3 months for those developing CNS disease versus 44.6 months for those with no CNS disease.^4^ However, studies with T-DM1 have reported less benefit in those patients with CNS disease as compared to those without CNS disease who received T-DM1, with TH3RESA reporting a median OS of 17.3 months (95% CI 10.3-26.2 months) and 23.7 months (95% CI 20.6-28.2 months), respectively, for patients with and without BM receiving T-DM1.^15^ An exploratory analysis of EMILIA reported a median PFS of 5.9 months for patients with CNS disease versus 9.6 months for all patients receiving T-DM1.^16^ DESTINY-Breast03 demonstrated that T-DXd was more active than T-DM1 in patients with stable BM^11^ (Supplementary Table S5, available at https://doi.org/10.1016/j.esmoop.2023.102033).

Our data are hypothesis generating and suggest that treatment with T-DXd in patients with BM results in such disease control that it is no longer a detriment to survival compared to patients with extracranial disease.^4^ Other data to support this hypothesis include supplementary data from the US real-world study which reported a longer 12-month overall PFS in patients with BM as compared to the overall population: 74.7% (95% CI 39.5% to 91.2%) versus 57.8% (95% CI 31.1% to 77.3%), respectively.^13^ DESTINY-Breast01 reported a median PFS of 18.1 months (95% CI 6.7-18.1 months) in patients with stable BM versus 16.4 months (95% CI 12.7 months-NE) in patients without BMs (HR 1.17, 95% CI 0.57-2.39)^12^ (Supplementary Table S5, available at https://doi.org/10.1016/j.esmoop.2023.102033). This intracranial therapeutic effect appears not to be limited to T-DXd, a pre-specified subgroup analysis of HER2CLIMB found in patients receiving tucatinib; the median OS was 21.6 months (95% CI 18.1-28.5 months) for those with BM versus 24.7 months (95% CI 21.6-28.9 months) for those without BM.^14^^,^^17^ However, HER2CLIMB and US real-world study did not present comparison between those with extracranial disease and those with active BM.^13^^,^^14^ Overall, these data suggest that BM treated in the era of second-generation ADCs and oral HER tyrosine kinase inhibitors has improved survival outcomes which are as good as in patients without BM.

The rate of pneumonitis in the current study was 31%; the majority were low grade but two related deaths occurred. No other real-world data on pneumonitis are currently available. The reported rate of pneumonitis in the most comparable trial population namely DESTINY-Breast02 was 6% (25) with two grade 5 deaths (<1%).^10^ The reasons for the higher rate of pneumonitis reported here are not clear, but may be related to underlying risk factors within our current population as well as the differences in reporting within DESTINY-Breast02 where all possible cases of pneumonitis were adjudicated by an independent committee,^10^ while the higher proportion of deaths is likely related to the early use of a new agent within a real-world setting, with a relative lack of awareness of the importance of recognising and treating pneumonitis as compared to the well-controlled environment of a clinical trial.

One patient had pathologically confirmed radionecrosis following commencing T-DXd. The patient had suspended T-DXd before the surgical debridement due to fatigue and has not since restarted. Only one other case of radionecrosis following T-DXd has been reported, which resulted in the discontinuation of treatment.^13^ Radionecrosis is a recognised adverse effect with T-DM1.^18^ Further work is required to understand the incidence and its implications of radionecrosis in patients receiving T-DXd.

Limitations of the current study include the data being retrospective although patients were identified from a prospective database and a lack of standardised assessment intervals for imaging. While the BM cohort was a small study, it was comparable to other studies.^8^^,^^9^^,^^13^ Differences existed between our BM and extracranial only population in terms of median time since the diagnosis of metastatic disease to commencement of T-DXd and proportion presenting with *de novo* disease. These differences may have impacted the differences of the reported outcomes. Data for haematological and biochemical toxicity were not available in 7 of 29 (24%), due to data access issues at peripheral clinics and so limits some of our toxicity reporting.

In conclusion, we demonstrate that in a contemporaneous, real-world population with advanced HER2-positive BC a significant number have BM at the time of commencing T-DXd, the vast majority of which are active. T-DXd has clinical activity that is similar in patients with and without BM; these observations warrant further investigations in a larger series with the ability to control for possible differences as well as further analysis of the pivotal DESTINY-Breast studies. The higher rate of pneumonitis reported warrants further consideration particularly to ensure education around the diagnosis and management of this specific toxicity.
