# Supplementary material for: A comparison of the efficacy of trastuzumab deruxtecan in advanced HER2-positive breast cancer: active brain metastasis versus progressive extracranial disease alone
Source: ESMO Open. 2023 Oct 20;8(6):102033. doi: 10.1016/j.esmoop.2023.102033 (PMC10774880; doi:10.1016/j.esmoop.2023.102033)
Supplement: Supplementary Tables [file mmc1.docx]

|  | **All**  **(n= 22)** | **Extracranial Cohort (n= 13)** | **CNS (n=9)** | | **Total BM Cohort**  **(n=8)** | **LMD (n=1)** |
| --- | --- | --- | --- | --- | --- | --- |
| **Number of prior lines of treatment for MBC; median (range)** | | | | | | |
| Endocrine | 0 (0-4) | 0 (0-3) | 0 (0-4) | 0 (0-4) | | 1 (1) |
| All prior * | 2 (2-10) | 2 (2-5) | 2 (2-10) | 2 (2-10) | | 3 (3) |
| **Prior endocrine therapy; n (%)** | | | | | | |
| Exemestane | 4 (18) | 2 (15) | 2 (22) | 1 (13) | | 1 (100) |
| Goserelin and letrozole | 4 (18) | 1 (8) | 3 (33) | 3 (38) | | - |
| Tamoxifen | 3 (14) | 2 (15) | 1 (11) | 1 (13) | | - |
| Fulvestrant | 3 (14) | 1 (8) | 2 (22) | 2 (25) | | - |
| Letrozole | 2 (9) | 2 (15) | - | - | | - |
| Everolimus | 1 (5) | - | 1 (11) | 1 (13) | | - |
| Goserelin and Exemestane | 1 (5) | - | 1 (11) | 1 (13) | | - |

**Supplementary Table 1: Summary of endocrine treatment where used as a line of therapy for patients with oestrogen receptor-positive disease**

BM: Brain metastases; CNS: Cranial nervous system; LMD: Leptomeningeal disease; MBC: Metastatic breast cancer.

*All prior is the combination of endocrine therapy, targeted therapy and chemotherapy where treatments were used as new lines of therapy, but excludes those commenced as maintenance therapy.

|  | **Overall (n=29)** | **Extracranial cohort (n=16)** | **CNS (n=13)** | **BM (n=12)** | **LMD (n=1)** |
| --- | --- | --- | --- | --- | --- |
| Median number of cycles; n (range) | 11 (1-26) | 10 (2-25) | 12 (1-26) | 13 (1-26) | 1 (1) |
| **Treatment interruption;** | | | | | |
| Total number of treatment interruptions; n | 31 | 15 | 16 | 16 | - |
| Number of patients with treatment interruptions, n (%) | 16 (55) | 8 (50) | 8 (62) | 8 (67) |  |
| **Reasons for treatment interruption** |  |  |  |  |  |
| Infection | 13 (45) | 7 (44) | 6 (46) | 6 (50) | - |
| Pneumonitis | 5 (17) | 3 (19) | 2 (15) | 2 (17) | - |
| Thrombocytopenia | 5 (17) | 2 (13) | 3 (23) | 3 (25) | - |
| Fatigue | 2 (7) | 2 (13) | - | - | - |
| Neutropenia | 2 (7) | - | 2 (15) | 2 (17) | - |
| Anaemia | 2 (7) | - | 2 (15) | 2 (17) | - |
| TIA | 1 (3) | 1 (6) | - | - | - |
| Osteonecrosis of the jaw | 1 (3) |  | 1 (8) | 1 (8) |  |
| **Dose reduction;** | | | | | |
| Total number of dose reductions; n | 14 | 8 | 6 | 6 |  |
| Number of patients with 1 dose reductions, n (%) | 12 (41) | 7 (44) | 5 (38) | 5 (42) | - |
| Number of patients with 2 dose reduction, n (%) | 2 (7) | 1 (6) | 1 (8) | 1 (8) |  |
| **Reasons for dose reductions** |  |  |  |  |  |
| Fatigue | 4 (14) | 2 (13) | 2 (15) | 2 (17) | - |
| Nausea | 3 (10) | 2 (13) | 1 (8) | 1 (8) | - |
| Diarrhoea | 2 (7) | 2 (13) | - | - | - |
| Pneumonitis* | 2 (7) | 1 (6) | 1 (8) | 1 (8) | - |
| Thrombocytopenia | 1 (3) | - | 1 (8) | 1 (8) | - |
| **Drug discontinuation; n (%)** | | | | | |
| Total | 15 (52) | 9 (56) | 6 (46) | 5 (42) | 1 (100) |
| Progressive disease and death | 10 (67) | 7 (78) | 3 (50) | 2 (40) | 1 (100) |
| Discontinuation due to toxicity | 5 (33) | 2 (22) | 3 (50) | 3 (60) | - |
| **Toxicity:** |  |  |  |  |  |
| Fatigue | 3 (20) | 1 (11) | 2 (33) | 2 (40) | - |
| Pneumonitis | 1 (7) | - | 1 (17) | 1 (20) | - |
| Nausea | 1 (7) | 1 (11) | - | - | - |

**Supplementary Table 2: Summary of median number of treatment cycles, dose interruptions, reductions, discontinuations and their reasons**

BM: Brain metastasis; CNS: Central nervous system; LMD: Leptomeningeal disease; TIA: Transient ischaemic attack.

* Grade 1 pneumonitisin both cases.

|  | **Overall (n=29)** | **Extracranial cohort (n=16)** | **CNS (n=13)** | **Total BM (n=12)** | **LMD (n=1)** |
| --- | --- | --- | --- | --- | --- |
| **Total cases; n (%)** | 9 (31) | 7 (44) | 2 (15) | 2 (17) | 0 |
| **Grade; n (%)** |  |  |  |  |  |
| 1 | 6 (21) | 5 (31) | 1 (8) | 1 (8) | - |
| 2 | - | - | - | - |  |
| 3 | 1 (3) | - | 1 (8) | 1 (8) | - |
| 4 | - | - | - | - |  |
| 5 | 2 (7) | 2 (13) | - | - | - |
| **Outcome of the event; n (%)** |  |  |  |  |  |
| Recommenced  T-DXd | 4 (44) | 3 (43) | 1 (50) | 1 (50) | - |
| Did not recommence  T-DXd* | 3 (33) | 2 (29) | 1 (50) | 1 (50) | - |
| Treatment-related death | 2 (22) | 2 (29) | - | - | - |

**Supplementary Table 3: Outcomes of documented cases of pneumonitis**

*Changed therapy due to progressive disease (n=1), grade 3 pneumonitis (n=1), and death from progressive disease (n=1).

|  | **Overall (n=8)** | **Extracranial cohort (n=5)** | **CNS (n=3)** | **Total BM (n=3)** | **LMD (n=0)** |
| --- | --- | --- | --- | --- | --- |
| Remained on T-DXd* | 2 (25) | - | 2 (67) | 2 (67) | - |
| Tucatinib, capecitabine and trastuzumab | 3 (38) | 3 (60) | - | - | - |
| Best supportive care | 3 (38) | 2 (40) | 1 (33) | 1 (33) | - |

**Supplementary Table 4: Summary of treatment post-radiological progression on T-DXd.**

* Low volume asymptomatic progression of CNS disease

| **Study** | **Protocol defined entry criteria with regard to CNS** | **Protocol mandated CNS Screening prior to study entry** | **Screen detected BM** | **BM patients as proportion of study population** | **Reported efficacy data in BM population** | **CNS progression events** |
| --- | --- | --- | --- | --- | --- | --- |
| DESTINY-Breast 01  Modi et al., 2020  Jerusalem et al., 2022 | **Excluded:**  BM that were untreated, symptomatic, or requiring therapy to control symptoms, any history of radiation, surgery, or other therapy, including steroids  or anticonvulsants, to control symptoms for BM  within 2 months of randomization/registration. | CT or MRI of the brain within 28 days of registration/  randomization | NR | 24 of 184 (13.0%) | **ORR:**  58% (95%CI 37–78)  **Median**  **PFS :**  18.1 months | **BM population:**  2 of 24 (8.3%)  **No BM population**  2 of 160 (1.3%) |
| **DESTINY-Breast02**  **Andre et al., (2023)** | **Included**  Clinically inactive BM.  Treated asymptomatic BM requiring no corticosteroids or anticonvulsants  **Excluded:**  Untreated and symptomatic BM, or those requiring therapy with corticosteroids or anticonvulsants to control associated symptoms. | CT or MRI of the brain within 28 days of randomization. | NR | **Trastuzumab**  **deruxtecan**  74 of 406 (18%)  **TPC**  36 of 202  (18%) | **Median PFS**  **Trastuzumab**  **deruxtecan**  13·9 months  (95% CI: 11·1–18·0)  **TPC**  5·6 months  (3·3–8·1) | NR |
| **DESTINY-Breast03**  Cortes et al., (2022) | **Included**  Clinically inactive BM.  Treated asymptomatic BM requiring no corticosteroids or anticonvulsants.  **Excluded:**  Untreated and symptomatic BM, or requiring therapy with corticosteroids or anticonvulsants to control associated symptoms. | CT or MRI of the brain within 28 days of randomization. | NR | **Trastuzumab deruxtecan**  62 of 262  (23.8%)  **Trastuzumab**  **emtansine**  52 of 263 (19.8%) | **Median PFS**  **Trastuzumab**  **deruxtecan**  15.0  (95% CI: 12.6–22.2)  **Trastuzumab**  **emtansine**  5.7  (95% CI: 2.9–7.1) | NR |

**Supplementary Table 5: Summary of CNS entry criteria and reported brain metastasis data within DESTINY-Breast Studies**.

CNS: Central nervous system; CT: computed tomography; MRI:Magnetic resonance imaging NR: Not reported; ORR: Objective Response Data; PFS: Progression Free Survival; TPC: Treatment of Physicians Choice

|  | **TUXEDO** | **DEBBRAH**  **(Cohort 3^¶^)** | **US multicentre real-world study**‡ | **The Clatterbridge Cancer Centre real-world study** |
| --- | --- | --- | --- | --- |
| **Population** | Histologically confirmed breast cancer, with radiologically confirmed metastatic disease showing new or progressing brain metastases despite local treatment. | Histologically confirmed HER2-positive MBC with progressing BMs after local treatment. | Metastatic breast cancer with stable or active BM. | Histologically confirmed HER2-positive MBC with radiological confirmed metastatic disease. |
| **No of BM patients** | 15* | 9 | 18 | 12 |
| **Median Follow up; months** | 12 months (95% CI: 8 months–nr) | 8.8 (Range 2.1-10.8) | 7 | 13.8 (95% CI:9.9 – unobtainable) |
| **Median age; median (range)** | 69 (30–76) | 53.0 (37–61) | 46 (35-69) | 50 (43-53) |
| **Prevalence of BM; %** | Not reported | Not reported | Not reported | 41 |
| **Number of prior lines of treatment for MBC; median (range)** | 2 (1-5) | ≥5 (1 ->5) | 4 (0-10) | 2 (2-6) |
| **Prior treatment for metastatic disease; n (%)**   - T plus P - T-DM1 - Trastuzumab duocarmazine | 15 (100)  9 (60) | 7 (78)  6 (67) | 11 (61)  15 (83) | 11 (92)  12 (100)  1 (8) |
| **Definition of active BM** | Newly diagnosed BMs OR BMs progressing after local therapy. | Progressing BMs after local therapy. | MBC with untreated or progressive BMs. | Untreated or progressing BMs after local therapy. |
| **Brain Metastasis; n (%)**   - Untreated - Progressive disease after local therapy - Progressive disease - Stable disease | 6 (40)  9 (60)  -  - | -  9 (100)  -  - | **  3 (17)  -  7 (39)  2(11) | 1 (8)  9 (75)  -  1 (8) |
| **Response Criteria utilised** | RANO-BM | RANO-BM | Modified RANO-BM | RECIST 1.1 |
| **Intracranial Objective Response Rate; % (95% CI)**   - ITT population - Per protocol population   **Clinical Benefit Rate:**   - ITT population - Per protocol population | 73.3 (48.1-89.1)  78.6 (49.2-95.3)  86.7 (13 of 15)  92.9 (66.1–99.8) | 44.4 (13.7-78.8)  77.8 (40.0-99.5) | **  73.3  Not reported | ***  58 (30-86)  83 (62-100) |
| **Median Progression free survival: Months (95% CI)** | 14 (11.0-NE) | Not reported | NR (7.0-NE) | 17.0 (15.2 -NE) |
| **12-month PFS: % (95% CI)** | Not reported | Not reported | 74.7 (39.5-91.2) | 88 (67-100) |
| **Median Overall Survival; Months (95% CI)** | NR  (3 deaths at 12 months median follow up) | Not reported | Not reported | NR (15.3-unobtainable)  2 deaths |
| **12 month OS; % (95% CI)** | Not reported | Not reported | Not reported | 88 (67-100) |
| **Extracranial only disease who progressed to get BMs; n (%)** | Not reported | Not reported | Not reported | 1 (8) |
| **Pneumonitis; n (%)** | 1 (7) | 3 (33) | Not reported | 1 (8) |
| **Radionecrosis; n (%)** | Not reported | Not reported | 1 (6) | 1 (8) **** |

**Supplementary Table 6: A summary and comparison of previous studies reporting activity of trastuzumab deruxtecan in brain metastasis to the Clatterbridge Cancer Centre cohort**

BM: Brain metastasis; CI: Confidence interval; ITT: Intention-to-treat; MBC: Metastatic breast cancer; NE:Not Estimable; OS: Overall survival; PFS: Progression-free survival; RANO-BM: Response assessment in neuro-oncology brain metastases; RECIST: Response evaluation criteria in solid tumours.

¶) Cohort 3: HER2-positive, advanced breast cancer with progressing brain metastasis after surgery, stereotactic radiosurgery, and/or whole brain radiotherapy.

‡ American multicentre study: Using total cohort characteristics. One patient was excluded from outcome analysis due to missing follow-up data. Two patients did not have confirmed HER2-positive BCBMs (One with no tissue diagnosis, one IHC 1+/FISH-negative).

*One patient found to have dural metastasis is included in response rate in the intention-to-treat population and the safety population, but excluded from further efficacy analyses.

** Out of the 15 patients with evaluable BMs. BM per investigator status not presented for 3 patients, however was used for ORR. Progressive disease not defined with regards to previous local treatment.

***In patients who were assessable at the time of reporting.

**** We report one patient who suffered radionecrosis. Patient had T-DXd held before surgical debridement due to fatigue and did not restart postoperatively.
